# Morphological changes during the post-embryonic ontogeny of mesothelan spiders and aspects of character evolution in early spiders

**DOI:** 10.1007/s00427-021-00675-y

**Published:** 2021-04-18

**Authors:** Thomas Huber, Carolin Haug

**Affiliations:** 1 LMU Munich, Biocenter, Großhaderner Str. 2, 82152 Planegg-Martinsried, Germany; 2 GeoBio-Center of the LMU Munich, Richard-Wagner-Str. 10, 80333 München, Germany

**Keywords:** *Ryuthela nishihirai*, Post-embryonic development, Heterochrony, Sexual dimorphism, Sternum

## Abstract

Most morphological studies focus on adult specimens, or if developmental studies are pursued, especially in Euarthropoda, they focus on embryonic development. Araneae (spiders) is one of these groups, in consequence with understudied post-embryonic development. Here we present aspects of the post-embryonic stages of different species of Mesothelae, sister group to the remaining spiders (when fossil species are not taken into account). We used different imaging methods and measured different external morphological structures to detect possible ontogenetic changes. One structure exhibiting post-embryonic changes is the chelicera. Here the significant change occurs between the last immature stage and the adult, yet only in males. For the spinnerets, we could not detect ontogenetic changes, but instead a high variability in length and width, probably due to their lack of pivot joints between the elements. The strongest morphological change during ontogeny occurred on the sternum, which begins with a rather roundish shape in the first stage and changes to being fairly elongate in shape in the last immature stages and the adult. This specific sternum shape only occurs in adults of mesothelan spiders, while opisthothelan spiders have a broader sternum also in the adult. We discuss our results in an evolutionary context, also taking into account recent finds of fossil spiders.

## Introduction

The majority of zoological research focuses on adult individuals (e.g. Minelli et al. 2006). As a result, the morphology of immatures and its differences from that of the conspecific adults remains largely unknown, especially (but not only) concerning the post-embryonic stages. One such animal group is Araneae, spiders.

Araneae is composed of the sister groups Mesothelae and Opisthothelae, at least from an extant view (for recent fossil finds, especially of early representatives of Araneae, see, e.g. Huang et al. 2018; Wang et al. 2018; and references therein). All extant species of Mesothelae occur in Southeast and East Asia (Haupt 2003). In the Carboniferous (c. 300 million years ago), spiders resembling modern representatives of Mesothelae were spread across Euramerica (Selden et al. 2014). Mesothelan spiders are medium to large size and live in burrows sealed with a door out of silk and soil and spend their entire life inside the burrow. Their prey is captured right in front of the burrows while close contact to the entrance is kept. Silk is used to build the burrow and the door, but no true net is spun. The only exceptions are representatives of the group *Liphistius* Schiödte, 1849 which use fishing lines to catch their prey (Haupt 2003).

The representatives of Mesothelae have retained numerous plesiomorphic features, even more so than mygalomorph spiders, or bird spiders, which they largely resemble on a first sight. One example for such a plesiomorphy is that the opisthosoma has clearly visible tergites on the dorsal side and sternites on the ventral side, which are not present in all other spiders. A characteristic of (adult) mesothelan spiders is a rather narrow sternum on the ventral side of the prosoma (Haupt 2003). So far it is unclear if its narrow shape represents an apomorphy or a plesiomorphy for Mesothelae.

The spinnerets of spiders arise from the 4th and the 5th opisthosomal segment (e.g. Yoshikura 1955; Kaestner 1965) and are localized in the middle of the regularly segmented opisthosoma in mesothelan spiders. Opisthothelan spiders have lost the external features of segmentation of the opisthosoma, and their spinnerets are in a terminal position.

As the spinnerets of mesothelan spiders are not in a terminal position, the opisthosoma cannot contribute much to their movability as it is the case in opisthothelan spiders. Instead, the spinnerets of mesothelan spiders have the highest number of elements of all spiders, providing them with a wide range of motion. At the time of hatching, seven of such elements are present (Yoshikura 1955). During the next moults, the number of elements increases to 9–12 elements. This stepwise increase of spinneret elements has been considered as an autapomorphy for Mesothelae (Haupt 2003). Opisthothelan spiders have spinnerets with only 3 or less elements. Additional to the high number of elements, mesothelan spiders have spinnerets supported by a complicated muscular system, which provides them with a high degree of flexibility (Abraham 1923). Pivot joints between the elements have never been mentioned for spinnerets, and the absence of true pivot joints could be another reason for the flexibility of the spinnerets.

Mesothelan spiders exhibit a pronounced sexual dimorphism. Male spiders of species of *Heptathela* Kishida, 1923, and *Ryuthela* Haupt, 1983, live about 5 years and die a few weeks after maturation (Haupt 1979, 1991; Song and Haupt 1984). Males often mature earlier than females and leave their burrow after the last ecdysis (Haupt 1986). Their morphology is different in several aspects compared to that of the females, reflecting their focus on mating rather than catching prey. They are younger at maturation and therefore smaller but have long walking legs, presumably as an adaption to their vagrant life, wandering around and looking for a mating partner (Haupt 2003). The chelicerae instead seem to be smaller in relation to body size. Female spiders, however, live up to ten (*Heptathela kimurai* (Kishida, 1920)) or even 20 years (*Ryuthela nishihirai* Haupt, 1983) and continue to grow during that time, in contrast to males which live only few weeks after reaching maturity (Haupt 1979, 1991; Song and Haupt 1984). Their walking legs are relatively shorter than those of the males, and they mainly stay in their burrows. The chelicerae remain at the same relatively large size as in immatures.

In 1977, Theodore Savory stated “Araneists may be well advised to abandon their traditional habit of neglecting or even throwing away immature specimens that they find, for these have much to teach us, and even the cast-off exoskeletons left after moulting may be profitably examined” (Savory 1977, p. 50). For this study, both exuviae and carcasses of different instars of mesothelan spiders have been investigated to reconstruct almost complete post-embryonic ontogenetic series of exemplary species. The changes of morphological characters during ontogeny have been analysed in detail and put into an evolutionary context.

## Material and methods

### Material

The specimens used in this study have been bred or collected by the late Joachim Haupt, Berlin. They are part of the collections of the “Zoologische Staatssammlung München” (ZSM). The entire collection of mesothelan spiders was inspected (several hundred specimens), and the best preserved specimens were included into the analyses (see below). Representatives of four different mesothelan species were investigated. The research mainly focused on *Ryuthela nishihirai*, but the results were compared with data collected from *Ryuthela ishigakiensis* Haupt, 1983, and *Heptathela kimurai* and *Liphistius batuensis* Abraham, 1923.

### Imaging

**Fig. 1:**
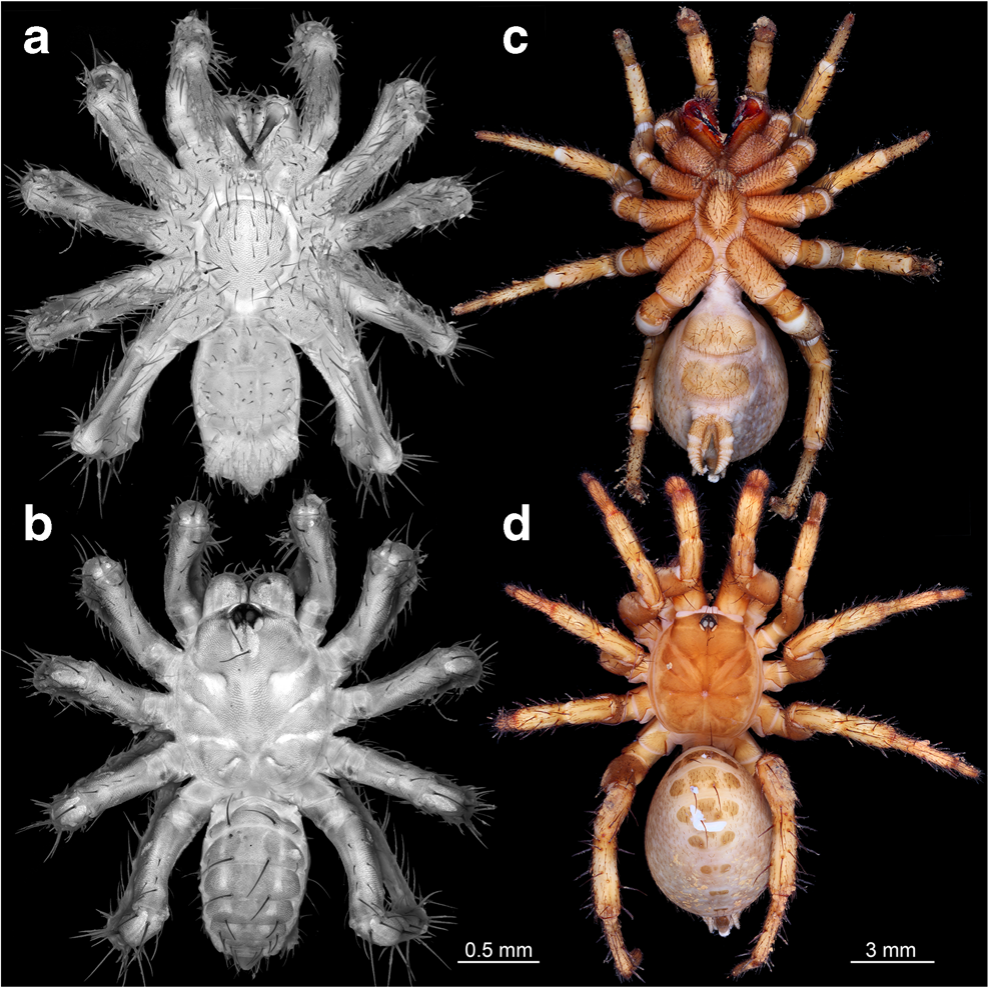
Different imaging methods, exemplified on specimens of *Ryuthela nishihirai.* **a**, **b** Immature stage II; combined images based on microscopy under autofluorescence and brightfield settings. **a** Ventral view. **b** Dorsal view. **c**, **d** Adult female; macrophotography under cross-polarised light. **c** Ventral view. **d** Dorsal view

Small specimens were documented using a Keyence BZ-9000 fluorescence microscope (Fig. 1 a and b). The objectives used were × 4, × 10 and × 20, resulting with the camera magnification in a total magnification of × 40, × 100 and × 200, respectively. The specimens were documented under both brightfield and autofluorescence settings as some structures (especially setae) were only visible under one of these settings. For autofluorescence, a GFP (blue/green) or TRITC (green/orange) filter set was used (excitation wavelength: 473 nm resp. 543 nm), depending on the properties of the specimen (Haug et al. 2011b).

Specimens too large for a proper documentation under the microscope, especially concerning z-axis, were documented with macrophotography (Fig. 1 c and d). A Canon Rebel T3i digital camera and a Canon MP-E 65mm macro lens with a polarisation filter were used. Two Yongnuo Digital Speedlite YN560EX II flashes with a polarisation filter were used to illuminate the specimen. As the specimens were documented in 70% ethanol, the cross-polarisation of the light was necessary to avoid reflections (e.g. Haug et al. 2011a).

To achieve entirely focused images, i.e. to overcome limitations in the depth of field, image stacks with continually shifting focus were recorded; adjacent image details were recorded to overcome limitations in the field of view (e.g. Haug et al. 2008; Haug et al. 2011a). The image stacks were fused using CombineZP. Fused images of adjacent areas were stitched using Microsoft ICE 2.0.3.0 or Adobe Photoshop Elements 11. The resulting compound images from brightfield and autofluorescence settings of the same specimen (see above) were combined using Photoshop Elements 11, similar to the processing described by Rötzer and Haug (2015) for different exposure times. Further image processing (contrast enhancement, removal of the background) was performed using Adobe Photoshop CS2. Drawings were produced in Illustrator CS2.

### Measurements

Measurements were carried out in ImageJ 1.50b. All measurements were normalized using the length of the prosomal shield as it is the largest sclerotized structure of the spider. Not all structures could be measured on all specimens as they were not always preserved: the lengths of the spinnerets were measured on 16 specimens of the species *Ryuthela nishihirai*. To analyse the sexual dimorphism in case of the chelicerae, 10 immatures, 5 female, and 3 male adult specimens of the species *Ryuthela nishihirai* were used. The broadest site of the chelicerae was measured, and the mean of both chelicerae was divided by the length of the prosomal shield. To characterize the sternum, 28 spiders of the species *Ryuthela nishihirai*, 6 specimens of the species *Ryuthela ishigakiensis*, 3 specimens of the species *Liphistius batuensis*, and 8 specimens of the species *Heptathela kimurai* were measured.

### Reconstruction of ontogenetic stages

As we did not breed the spiders but used museum material, we deduced the immature stage from the length of the prosomal shield. In *Ryuthela nishihirai*, individuals in stage I start with a prosomal shield of about 0.8–0.9 mm length. Adult spiders are about six times larger. In general, there are eight stages until the spider becomes adult (Haupt 1986, 2003).

## Results

### General ontogenetic aspects

**Fig. 2:**
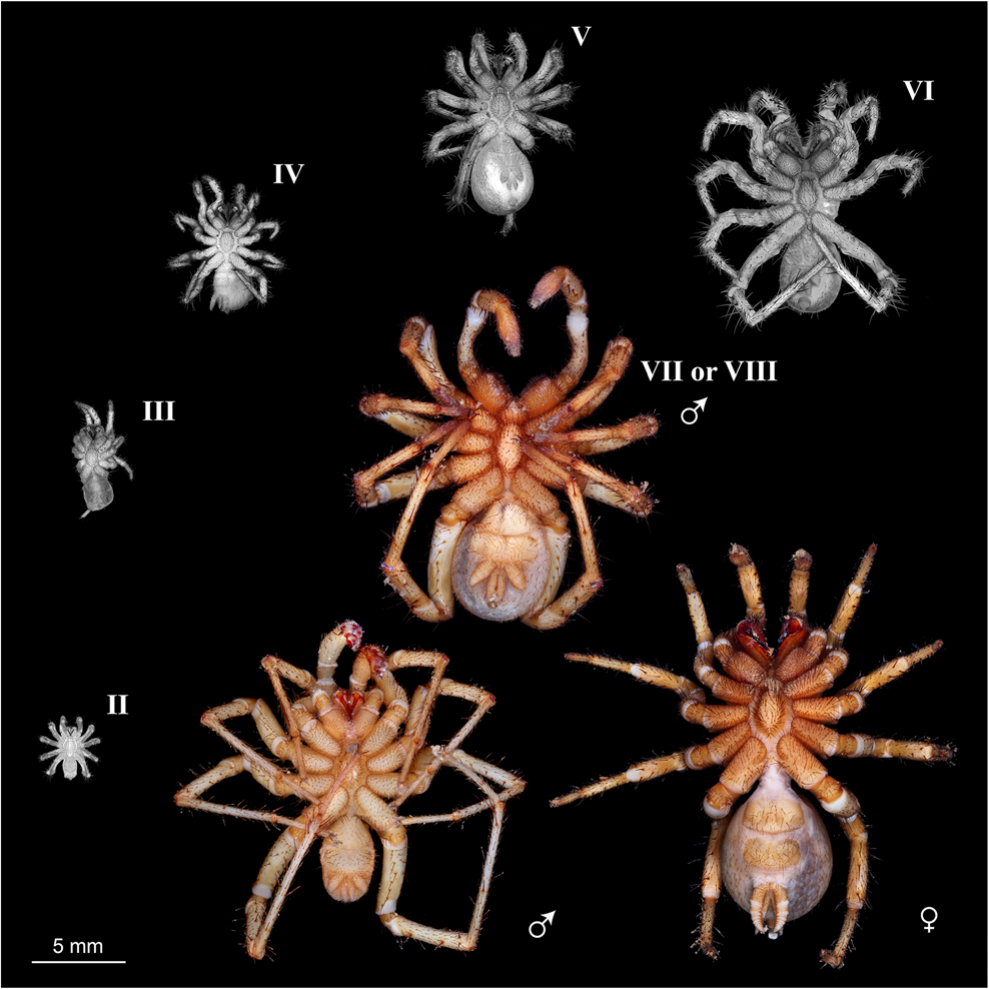
Ontogeny of *Ryuthela nishihirai*, all specimens to the same scale. Roman numerals indicate the immature stage of the spiderling. Spiders without a Roman numeral are adult. Grayscale images were taken via fluorescence microscopy using a GFP fluorescence filter set to capture the autofluorescence of the specimens. Colour images were taken via macrophotography using cross-polarised light. The material contained no carcass in stage I (but this stage was represented among the exuviae), no complete spiderling in stage VI and no complete subadult individual in stage VII–VIII (walking leg or chelicerae missing)

We were able to distinguish six post-embryonic immature stages in *Ryuthela nishihirai* (Fig. 2). The so-called post-embryo (after Downes 1987 and Wolff and Hilbrant 2011), which hatches from the egg, was not observed. Major changes in the morphology of the spiderling occur before immature stage III (e.g. development of eyes, trichobothria) and during the transition from the penultimate stage (usually called subadult) to the adult stage, resulting in the sexual dimorphism in the adults.

Hatchlings of *Heptathela kimurai*, a close relative of *R. nishihirai*, are blind and develop eyes within the next moult (Haupt 1986). As the smallest carcass of *R. nishihirai* in our material had fully developed eyes and smaller exuviae were available, we concluded that this specimen is in stage II, and stage I is missing among the carcasses (Fig. 2) but present among the exuviae.

An additional problem was that immature stages can be skipped. The skipping of stages is more frequent in males than in females (Haupt 1986). Hence, the subadult male spider in our material could be in stage VII or VIII (Fig. 2).

### Chelicera

**Fig. 3:**
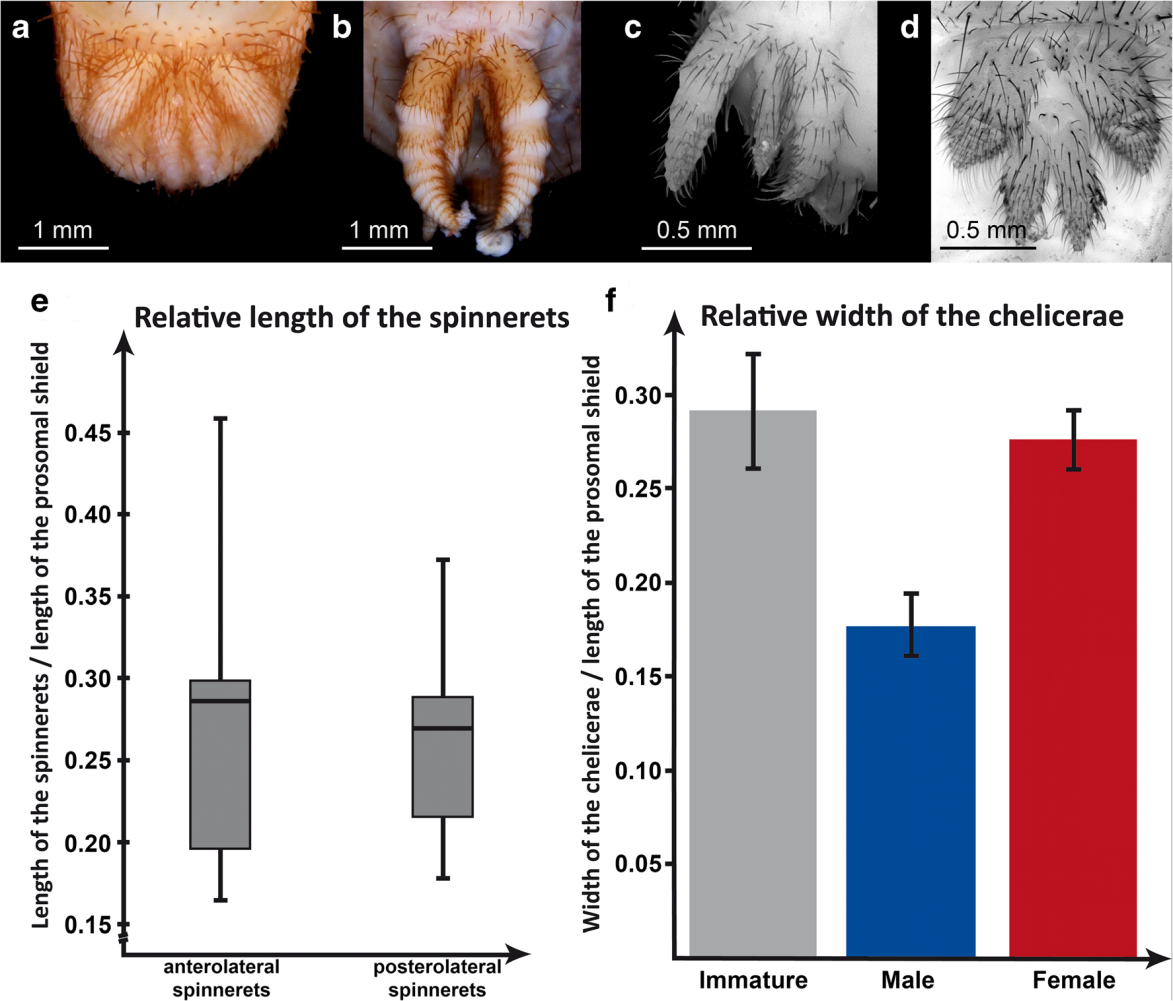
Intraspecific variability of spinnerets and chelicerae of *Ryuthela nishihirai*. **a**–**e**. Variability of spinnerets. **a** Adult male, macrophotography. **b** Adult female, macrophotography. **c**, **d** Immatures, autofluorescence microscopy. **e** Relative length of the different spinnerets, indicated by the ratio of the length of the spinnerets divided by the length of the prosomal shield. The box plot indicates the high variability within the material. **f** Relative width of the chelicerae, indicated by the ratio of the width of the chelicera and the length of the prosomal shield. Immatures have a ratio around 0.29, very similar to that of adult females (0.28), but apparently more than adult males (0.18). The standard error is indicated by the black line

The strongly expressed sexual dimorphism between male and female mesothelan spiders becomes apparent from the longer walking legs in males (compare adult male and adult female in Fig. 2) but also from the different dimensions of the chelicerae. During the moult to the adult, male spiders appear to develop smaller chelicerae. Immatures exhibit a ratio of width of the chelicerae versus length of the prosomal shield of about 0.29 (Fig. 3f). This ratio appears to be not significantly different to that of adult females as they retain the characteristic massive chelicerae. Adult males instead appear to reduce the size of the chelicerae to a ratio of 0.18 (Fig. 3f).

### Spinnerets

It was not possible to correlate the size of a spider with the length of its spinnerets. All tested mesothelan species exhibited huge variation in the length of the spinnerets relative to body size (Fig. 3e). Not just the length but also the shape of the spinnerets varied significantly from specimen to specimen. Some were short but broad and others longer but slender.

Neither macrophotography nor fluorescence microscopy of the spinnerets revealed any sign of pivot joints, which usually connects the different true appendage elements. This indicates that mesothelan spiders do not have true pivot joints in their spinnerets (Fig. 3a–d).

### Sternum

**Fig. 4:**
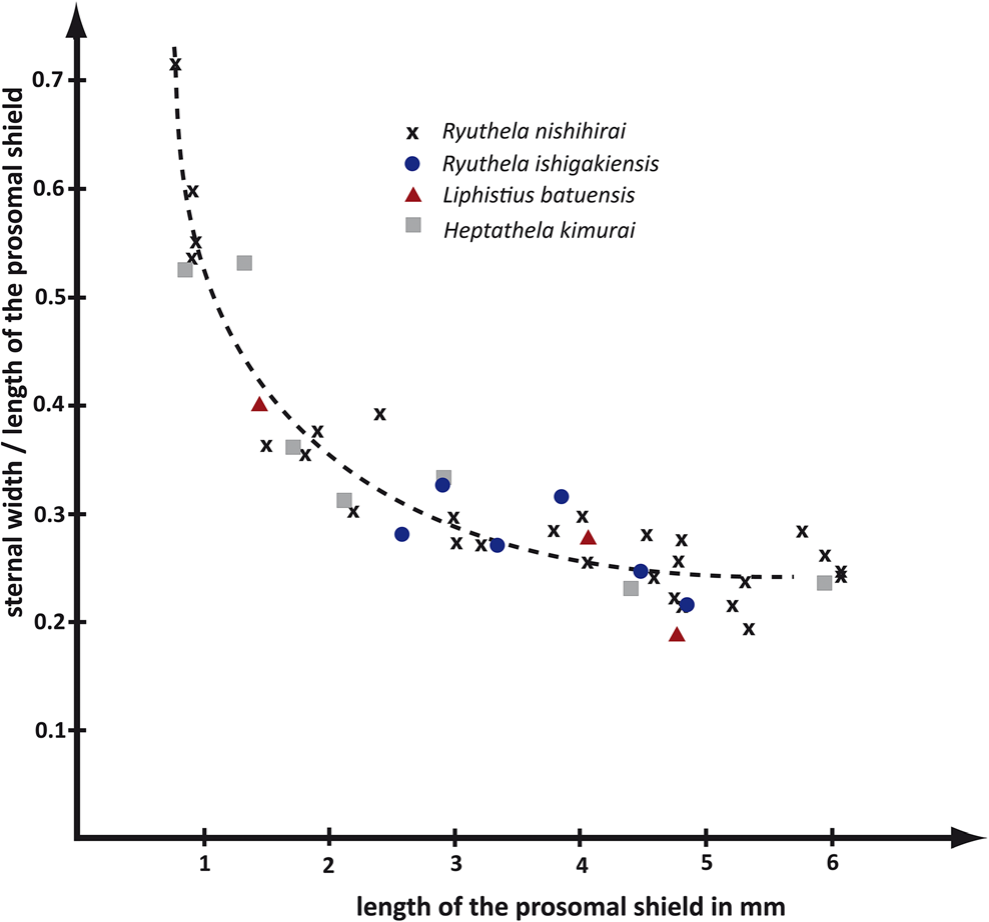
Apparent relative narrowing of the sternum during ontogeny, measured on four different mesothelan species of about the same adult body size. Specimens with a prosomal shield longer than 4.5 mm are considered adult

**Fig. 5:**
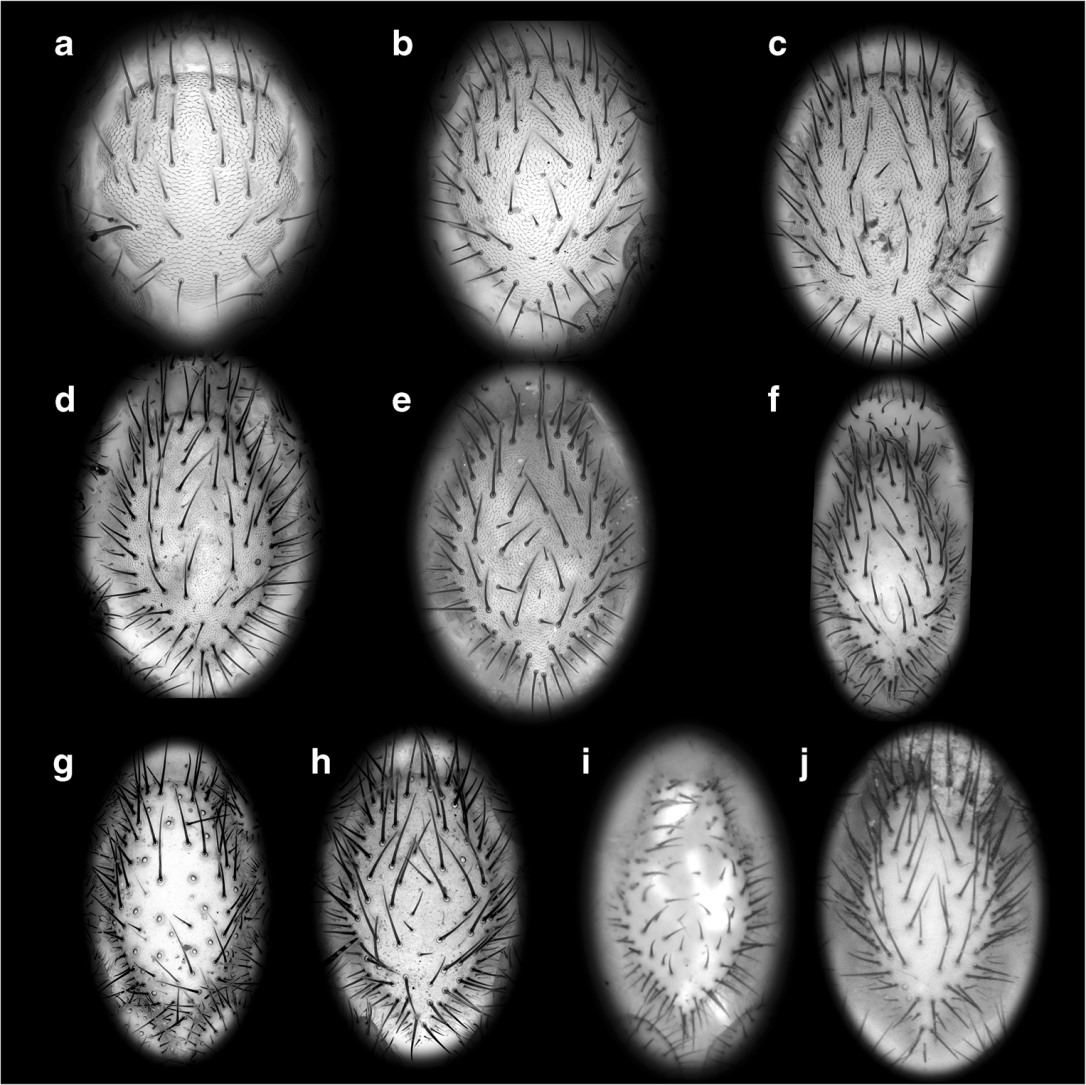
Sterna of different ontogenetic stages of *Ryuthela nishihirai*. Sterna depicted at the same length, but not to scale to show the apparent progressive narrowing during ontogeny. **a**–**f** Immatures sorted according to the length of the prosomal shield (immature stages II–VI). **g** Subadult male. **h** Subadult female. **i** Adult male. **j** Adult female

During post-embryonic ontogeny, the sternum of the four species of Mesothelae investigated in this study appears to become relatively narrower (Figs. 4 and 5). At stage I, the animals have a broad and rounded sternum, which is about 0.5 mm long and 0.5 mm wide. Very small specimens at stage I even have a sternum which is slightly wider than long.

The adult sternum is more than twice as long as wide (in our study, most spiders with a length of the prosomal shield longer than 4.5 mm were considered to be adult). The actual size varies as female spiders continue to grow also after reaching adulthood. The sternum becomes also relatively shorter in adults compared to body size, but the change is not as drastic as the change in width: the ratio of the sternal length versus the length of the prosomal shield decreases from about 0.68 (maximum) to 0.39 (minimum) during ontogeny. The ratio of the sternal width versus the length of the prosomal shield, however, decreases from about 0.71 (maximum) to 0.19 (minimum) (Fig. 4). According to the investigated material, it takes about 4 or 5 moults after hatching until the sternum reaches the adult shape which stays about the same in the subsequent moults (Fig. 5).

## Discussion

We found different morphological structures that differ between the specimens. These differences appear to be correlated with differences in overall body size. We therefore interpret these changes as coupled to post-embryonic ontogeny. It is important to note that the results are based on a limited amount of specimens which were well enough preserved to be measured; thus no strict statistical tests could be applied. However, especially the ontogenetic changes of the sternum appear to follow a distinct trajectory for all investigated specimens (Fig. 4); hence these changes appear to be real ontogenetic changes.

### Chelicerae

Male adults of the mesothelan species *Ryuthela nishihirai* appear to have smaller chelicerae than conspecific female adults and immatures (Fig. 3f). This observation is coherent with the life history of male and female mesothelan spiders: females can live up to 10 or even 20 years, while males die a few weeks after maturation. Therefore, the adult males do not need as efficient preying abilities as the females. Whether male spiders completely stop eating or if they just focus on smaller prey is not known; both would be possible. According to Schwendinger (pers. comm., cited from Foelix and Erb 2010), males of the group *Liphistius* hardly capture any prey after reaching adulthood. Furthermore, it is still not clear if adult mesothelan males have venom glands. Haupt (2003) claimed that mesothelans in general have no venom glands (in contrast to opisthothelan spiders), but Foelix and Erb (2010) were able to show the opening of the venom glands on the chelicerae in different species of *Liphistius* as well as the venom gland itself. Yet, the lack of these structures mentioned by Haupt (2003) might be a misinterpretation from investigating only males (see discussion in Foelix and Erb 2010).

Another possible interpretation for smaller chelicerae in adult males is their position next to the pedipalps. Smaller chelicerae could therefore leave more space for the movements of the pedipalps. As male spiders copulate with their pedipalps, this might ease copulation and act as a selection pressure for the reduction of the size of the chelicerae in adult males, independent of the feeding behaviour of the male spiders.

In general, the smaller chelicerae as well as the longer walking appendages in the male mesothelan spiders could be the result of a heterochronic event only affecting males: the female in principle retains the morphology of the late immature stages, while males indeed add a new morphology at the end of their ontogeny in a peramorphic event, most likely representing a case of hypermorphosis (for details on heterochrony, see Webster and Zelditch 2005). The females therefore retain more plesiomorphic morphological characters, while males possess more derived ones. This kind of sexual dimorphism is so far unknown for the supposed close relatives of spiders, namely, whip spiders (Amblypygi) or whip scorpions (Uropygi = Thelyphonida + Schizomida) (together forming the monophyletic group Megoperculata). Yet, at least the longer walking legs in male adults might already be part of the ground pattern of Araneae as it is known also for several mygalomorph spiders (e.g. Grossi et al. 2016).

While it may seem counterintuitive to have heterochronic events only affecting one sex, this is in fact not unusual. In different lineages of beetles (Coleoptera; fire flies, trilobite larvae), the females have become affected by paedomorphosis, in principle retaining larval morphology, while males gain “normal” adult morphology (e.g. Fu et al. 2012). In isopodan crustaceans of the group Gnathiidae, the female adults largely resemble the larval stages with a rather “typical” isopodan-type morphology. In contrast, the adult males have an aberrant morphology with enlarged heads and mandibles (e.g. Wägele 1987).

However, it would be necessary to evaluate the ontogeny of further opisthothelan spiders and representatives of Amblypygi and Uropygi for such morphological changes to achieve a more reliable character polarisation for reliably identifying heterochrony in the evolution of mesothelan spiders.

### Spinnerets

The spinnerets of mesothelan spiders arise from the middle of the opisthosoma in contrast to the spinnerets of opisthothelan spiders, which sit at the posterior end of the opisthosoma. Additionally, the spinnerets of mesothelan spiders consist of more elements than in any opisthothelan spider.

**Fig. 6:**
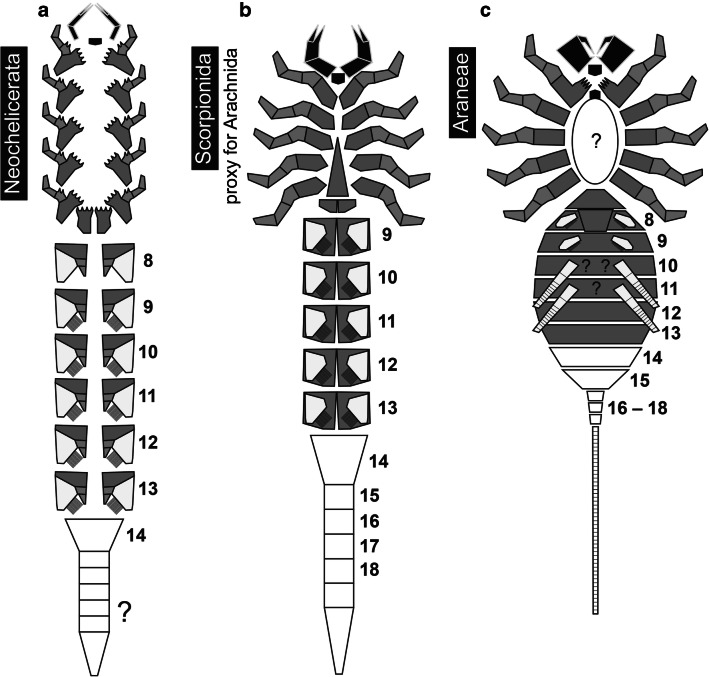
Condition of corresponding appendages in the evolutionary history towards the spinnerets in Araneae, illustrated with the reconstructed ground pattern conditions of different groups. **a** Ground pattern of Neochelicerata (Haug et al. 2019), closely resembling the condition in xiphosuridans. **b** Ground pattern of Scorpionida (= Scorpiones + fossil scorpions), probably closely resembling that of Arachnida. **c** Ground pattern of Araneae, based on the condition in Mesothelae and *Chimerarachne yingi* Wang et al. 2018. Numbers indicate post-ocular segment, question marks indicate unclear evolutionary origin and/or exact condition of certain structures. Colour coding: black = appendages of first post-ocular segment (chelicerae) and hypostome (“labrum”); dark grey = basipod; light grey = endopod; white = exopod and (possibly) limbless segments

Recently, the spider *Chimerarachne yingi* Wang et al. 2018, from the Cretaceous was found that retained the plesiomorphic “tail” known from other megoperculatans (Wang et al. 2018; see also Huang et al. 2018). The authors state that in this species, the spinnerets are in a more opisthothelan-like position. Yet, comparing the spinnerets position in *C. yingi* to that in mesothelan spiders reveals that the relative position is in fact almost identical, only the posterior three segments of the fossil species are more slender than in mesothelan spiders. This corroborates that a non-posterior position of the spinnerets is the ancestral condition for spiders, retained in mesothelan spiders (Fig. 6c).

We could not detect any significant changes of the morphology of the spinnerets during ontogeny. This is probably caused by a variability of the overall length, i.e. the elements or ringlets of the spinnerets appear to be able to partly telescope. This would only be possible if there are no joints, i.e. pivot joints, interconnecting these elements.

Whether the spinnerets originated from joint-bearing appendages has been discussed extensively (though without any focus on the presumed loss of the joints; e.g. Jaworowski 1896; Yoshikura 1955; Marples 1967; Shultz 1987; Damen et al. 2002; Hilbrant 2008; Selden et al. 2008; Pechmann and Prpic 2009; Clarke et al. 2015; Sharma 2017; recently reviewed in Mariano-Martins et al. 2020). Also if an evolutionary derivation from such a joint-bearing appendage is assumed, a still open question remains which parts of the appendage the spinnerets correspond to (see Fig. 6 for the morphology of the corresponding appendages in the evolutionary history towards Mesothelae; see also Haug et al. 2013 for the different appendage parts and appendage evolution).

Already earlier in the evolution towards spiders, the appendage derivatives on the corresponding segments did not possess distal appendage parts that have been suggested as the structure of origin (endopod, epipod); hence an origin from such structures for spinnerets is unlikely. Book lungs were ancestrally present on these segments as highly derived appendages (Fig. 6b), the appendage-like appearance of spinnerets is therefore either a de novo phenomenon or a reversal in the sense of a reactivation of an older morphology. In any case, if we want to draw a comparison to appendage derivatives, the lateral spinnerets could well be understood as correlating to exopods at best (Fig. 6c). The median spinnerets could, however, not easily be understood as endopods, as these structures have long been lost in evolutionary history. Also structurally and from developmental timing such an interpretation is not supported. The late appearance after the appearance of the lateral spinnerets (Yoshikura 1955) and the arising from a single membrane together with the lateral spinnerets (at least in different araneomorphan species; Machado 1944) could even be seen as an indication that these are not axial structures, but abaxial ones (see also anterior median spinnerets in Fig. 3D). Median abaxial structures seem to be present in the embryology of early representatives of spiders during their early ontogeny although they are absent in the adults (Pechmann and Prpic 2009).

### Sternum

The rather narrow sternum is characteristic of adult mesothelan spiders (Haupt 2003) and unique among spiders. However, our results indicate that this specific sternum morphology is not yet present at hatching and just develops post-embryonically. In all four investigated species, stage I individuals appear to possess a wide sternum which narrows in the subsequent moults. At a certain state the shape of the sternum apparently remains almost the same when the mesothelan spider moults the next time.

Haupt (1986) counted eight stages in the post-embryonic development of *Ryuthela nishihirai*. As we sorted the sterna according to the length of the prosomal shield of the specimens and there is a variance in size between spiderlings of the same stage, it is possible that one stage is shown two times and another one is missing. Most probably immature stages II–VI (Fig. 5a–f), male and female subadults (Fig. 5 g and h) and adults (Fig. 5 i and j) are represented in the investigated material.

The possible advantages of a narrow sternum are not easy to evaluate. When a mesothelan spider moults, it must enlarge the burrow. By narrowing the sternum, the basipods (termed coxae in arachnid terminology) of the walking legs are positioned more medially on the prosoma. This might relatively “shorten” the legs and could help the animal to fit into the old burrow for a longer time. However, we could not observe large differences in the relative length of the legs of immatures and adult females.

The arachnid ground pattern contains an endosternum, an internal sclerotisation in the prosoma present in xiphosuridans and most representatives of Arachnida (e.g. Snodgrass 1952). All basipodal (“coxal”) muscles are either attached to the endosternum or to the prosomal shield (Shultz 2007). No muscles of the walking legs are attached to the sternum. In Arachnida, the sternum is not primarily involved in the movement of the walking legs (with the possible exception in Araneomorpha, see Runge and Wirkner 2019). It is passively used to increase the hydrostatic pressure, which acts like an extensor muscle; the latter is missing in the ground pattern of Arachnida (Wilson and Bullock 1973; Stewart and Martin 1974; Anderson and Prestwich 1975; Prestwich 1988; Paul et al. 1989; Shultz 2007). Therefore, the shape of the sternum appears to have only little impact on the locomotion of mesothelan spiders.

**Fig. 7:**
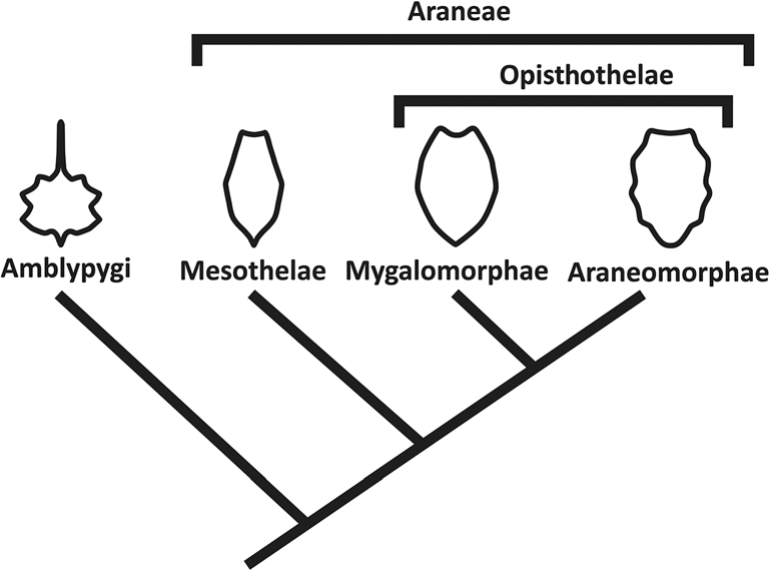
Phylogram of Amblypygi + Araneae with a schematic drawing of the adult condition of the sternum of each group

Hence, the mechanical consequences of a narrow sternum are not clear, but the development of the sternum in mesothelan spiders could shed some light on the evolution of early spiders. Opisthothelan spiders do not have a narrow sternum, neither as an immature nor as an adult. An outgroup comparison is necessary to shed light on the evolution of the sternum shape. One presumed sister group of Araneae is Amblypygi (Fig. 7, e.g. Paulus 2004 and references therein, though the internal relationships of Megoperculata are not settled yet; see above). Yet, the sternum of Amblypygi is quite different from that of Araneae. It is very small, about as long as broad, and has a long, anteriorly pointing spine, the so-called tritosternum (e.g. Shultz 1999). Hence, Araneae appear to have evolved a larger sternum compared to Amblypygi. Considering the character conditions, there are two possibilities for the evolution of the sternum in Araneae:

1. The narrow sternum already exists in the ground pattern of Araneae. Opisthothelae then developed a broader sternum. This could have happened through a heterochronic (in this case paedomorphic) event, resulting in a broader sternum in adult opisthothelans, resembling the condition in immature mesothelans.
2. A broad sternum is part of the ground pattern of Araneae, plesiomorphically retained from the last common ancestor of Amblypygi + Araneae. The narrow sternum is apomorphic for Mesothelae and evolved through a heterochronic (in this case peramorphic) event. Opisthothelae retained the ancestral, broader shape of the sternum.

However, the information about the ancestral condition of Amblypygi + Araneae is insufficient in this aspect. The difference in sternum morphology between Araneae and Amblypygi is too large to allow clear conclusions about the possible evolution of the sternum in araneaens.

Also the new fossils from the Cretaceous are inconclusive in this aspect. In the specimens shown in Wang et al. (2018), the sternum is not well preserved. In the specimens presented by Huang et al. (2018), the sternum is in fact different from that of both opisthothelan and mesothelan spiders. It appears narrower than that of opisthothelan spiders, yet not as narrow as in mesothelan spiders. Also, the mesothelan sternum has quite some distance to the basipods of the walking legs, which is not the case in the fossils. While in opisthothelan spiders the sternum appears to be also in closer contact to the basipods (especially in Araneomorpha, Runge and Wirkner 2019), the sternum shows distinct indents corresponding to the insertions of the legs. No such indents are apparent in the fossils. An ideal solution for the complicated situation would be finding fossils that allow the reconstruction of an ontogenetic series of tailed spiders.

## Data Availability

All data is provided in the text and the figures.
